# Temozolomide-induced increase of tumorigenicity can be diminished by targeting of mitochondria in *in vitro* models of patient individual glioblastoma

**DOI:** 10.1371/journal.pone.0191511

**Published:** 2018-01-19

**Authors:** Doreen William, Madlin Walther, Björn Schneider, Michael Linnebacher, Carl Friedrich Classen

**Affiliations:** 1 University Children’s and Adolescents’ Hospital, University Medicine of Rostock, Rostock, Germany; 2 Institute of Pathology, University Medicine of Rostock, Rostock, Germany; 3 Department of Surgery, University Medicine of Rostock, Schillingallee Rostock, Germany; Northwestren University, UNITED STATES

## Abstract

Glioblastoma multiforme (GBM) is a highly heterogeneous and aggressive brain tumor with a dismal prognosis. Development of resistance towards cytostatic drugs like the GBM standard drug temozolomide is a severe problem in GBM treatment. One potential source of GBM relapse could be so called cancer stem like cells (CSCs). These represent an undifferentiated subpopulation of cells with high potential for tumor initiation. Furthermore, it has been shown that differentiated GBM cells can regain CSC properties when exposed to continuous temozolomide treatment *in vitro*. In this study, treatment of several primary GBM cell lines with clinically relevant doses of temozolomide increased their tumorigenicity as determined by colony formation assays in soft agar. Increased tumorigenicity is a known property of CSCs. Hence, therapy options that specifically target CSCs are under investigation. CSCs appear to be particularly dependent on mitochondria biogenesis which may represent a useful target for CSC elimination. Toxicity towards mitochondria is a known side effect of several antibiotics. Thus, addition of antibiotics like doxycycline may represent a useful tool to inhibit CSCs in GBM. Here, we show that combining temozolomide treatment of primary GBM cells with doxycycline could counteract the increase of tumorigenicity induced by temozolomide treatment.

## Introduction

Glioblastoma multiforme (GBM) is a highly malignant brain tumor associated with a median survival of only 12 to 15 months despite aggressive multimodal standard treatment, consisting of resection followed by radio- and chemotherapy [1,2]. The development of resistance towards cytostatic drugs like the first line drug temozolomide is a problem for GBM treatment, and relapses of the disease are common [3]. One potential source of resistance and relapses are the so-called cancer stem-like cells (CSCs) within those tumors [4,5]. CSCs are a subpopulation of cells with the capability of self-renewal, increased drug resistance and potential of tumor (re-)initiation [6–8]. CSCs show expression of proteins associated with a physiological stem or progenitor cell state, for example CD133, CD15 and nestin [9]. The higher drug resistance of CSCs appears to be attributable to several different factors. There is evidence that CSCs show increased expression of MGMT [10] and multidrug resistance related proteins (MDRs) [11]. Furthermore, tumor cells can undergo dedifferentiation processes to acquire a CSC phenotype [12]. It has been shown that clinically relevant doses of temozolomide (TMZ) lead to enrichment of CSCs in a non-CSC population *in vitro*, which is not only caused by selection processes but also by conversion of non-CSCs into CSCs [12]. Hence, the direct targeting of CSCs might be a promising strategy for GBM treatment.

An Achilles heel of CSCs in several different tumor entities seems to be their increased dependence on mitochondria [13,14]. Mitochondria are essential for a variety of tumor functions [15]. Altered mitochondria function in cancer can lead to increased proliferation of tumor cells, apoptosis inhibition and induce a switch from catabolic to anabolic state [15]. It was reported that especially CSCs show an anabolic metabolism, supporting tumor initiation and proliferation [13].

Toxicity towards mitochondria is a known side effect of several antibiotics including tetracyclines [14]. Tetracyclines inhibit bacterial protein biosynthesis by reversibly binding the aminoacetyl-tRNA of the 30S subunit of 70S ribosomes. The 30S subunit of bacterial ribosomes is structurally very similar to the 28S subunit of mitochondrial ribosomes, enabling tetracyclines to bind and inhibit mitochondrial function [14]. Previous studies showed that treatment of GBM cells with doxycycline (Dox), a tetracycline derivate, inhibits spheroid formation of GBM-CSCs *in vitro*, which may indicate CSC inhibition [14]. Furthermore, CSCs of several tumor entities displayed an increased susceptibility towards treatment with antibiotics that are toxic towards mitochondria, compared to non-CSCs of the respective tumor entities *in vitro* [14].

In this study, four patient derived primary GBM cell lines were analyzed with regard to tumorigenicity upon TMZ treatment. We could show that continuous treatment of non-CSCs with therapeutic doses of TMZ lead to increased tumorigenicity *in vitro*, which can be counteracted by combined treatment with Dox.

## Materials & methods

### Cell culture

The primary GBM cell lines used in this study were previously established from patient individual GBM tissue in our laboratory [16]. Specimen collection was conducted in accordance with the ethics guidelines for the use of human material, approved by the Ethics Committee of the University of Rostock (Reference number: A 2009/34). Cells were cultured in DMEM/Ham’s F12 (Biochrom, Berlin, Germany) supplemented with 10% FCS (PAN-Biotech, Aidenbach, Germany) and 2mM L-Glutamine (Biochrom) and cultivated at 37°C, 5% CO_2_ and 95% relative humidity.

### Treatment with temozolomide and doxycycline

TMZ (MSD SHARP & DOHME GMBH, Haar, Germany) stock and Dox (AppliChem, Darmstadt, Germany) solutions were prepared at a concentration of 175mM (TMZ) and 39mM (Dox) in DMSO, aliquoted and stored at -30°C. Cells were treated with TMZ (50μM) alone, Dox (50μM) alone or a combination of both drugs (50μM each) for 2 cycles of 72h each. After 72h the medium was changed to freshly prepared medium containing the respective drugs and the cells were incubated for another 72h. Control cells were treated with DMSO only. Determination of cell viability after treatment was done by Calcein AM assay (Thermo Fisher Scientific).

### Colony formation assay

To determine the tumorigenic potential of the primary GBM cell lines, colony formation assays were performed in soft agar [17]. 96-Well plates were coated with 0.4% Agar dissolved in medium (100μl per well) and allowed to solidify.

Cells were harvested by trypsination and resuspended in 0.35% Agar dissolved in medium tempered to 40°C in a water bath. The cells were seeded in the pre-coated 96-well plates at a concentration of 500 cells per well and allowed to solidify. The cells were fed regularly by addition of 50μl medium per well and cultivated in a CO_2_ incubator at 37°C. After 14 days, the cells were stained with calcein AM viability dye (Thermo Fisher Scientific) and formed colonies were counted using an Elispot-reader (CTL Europe GmbH, Bonn, Germany).

### Western blot

Proteins were isolated from GBM cell lines by lysis of GBM cells with ripa buffer supplemented with protease inhibitor cocktail (Roche, Basel, Switzerland) following manufacturer’s instructions. Protein concentrations were determined via BCA assay kit (Merck Millipore, Massachusetts, USA). 30μg of protein per sample were separated on 8% SDS-polyacrylamide gels and subsequently transferred on polyvinylidene fluoride membranes using a tank blot system with Towbin buffer (25mM Tris, 192mM Glycine, 10% methanol, 0.1% SDS). After transfer, membranes were incubated in blocking solution (5% dry milk in tris buffered saline, pH 7.4) for 2h at room temperature (RT). Afterwards membranes were incubated with primary antibodies against CD15 (immunotools; 1:1000), nestin (Merck Millipore, 1:1000) and β-actin (Abcam, 1:1000) diluted in blocking solution for 2h. Prior to detection membranes were incubated with horseradish peroxidase-conjugated secondary antibodies (rabbit α-mouse-hrp, antibodies-online; 1:1000 in blocking solution) for 1h at RT. Detection was done with the Super Signal West Femto Kit (Thermo Fisher scientific) in a GelLogic 1500 Imaging System (Kodak, Berlin, Germany). Quantification was done by densitometric measurements of the digital images using ImageJ software [18]. The values were obtained by measuring the brightness of the western blot signal and the same area size was used throughout one blot.

As Tubulin was used as control, the values obtained for CD15 and Nestin were divided by the corresponding Tubulin values. To obtain the relative expression after treatment, the quotients of the treated samples were divided by the quotients of the untreated samples.

### Immunofluorescence staining of mitochondria

The cells were grown on cover slips in 24-well plates at 5000 cells per well and treated with temozolomide (50μM), doxycycline (50μM) or a combination of both drugs for 2 cycles at 72h each. For fluorescence staining of mitochondria the MITO-ID Red Detection Kit (Enzo) was used following manufacturer’s instructions.

### Quantification of mitochondria content

The relative amount of mitochondria in cell lines was determined by quantitative PCR. Therefore, total genomic DNA was isolated using the Genomic Wizard DNA extraction kit (Promega, Mannheim, Germany) following manufacturers’ instructions. 30ng of genomic DNA were used as template for quantitative PCR on a StepOne Plus Realtime PCR system (Applied Biosystems, Darmstadt, Germany) with SensiFastSYBR Hi-Rox-Kit (Bioline, Luckenwalde, Germany). Primers specific for regions that are only present in the mitochondrial genome (tRNA-Leu(UUR); forward: 5’-CACCCAAGAACAGGGTTTGT-3’, reverse: 5’-TGGCCATGGGTATGTTGTTA-3’) and primers specific for B2-microglobulin (forward: 5’-TGCTGTCTCCATGTTTGATGTATCT-3’, reverse: 5’-TCTCTGCTCCCCACCTCTAAGT-3’), which is specifically located on nuclear DNA were used [19].

### Analysis of MGMT promoter methylation

Analysis of MGMT promoter methylation was done using the MethyLight method [20]. Briefly, genomic DNA was subject to bisulfite conversion using the Epitect Bisulfite Kit (Qiagen, Hilden, Germany) according to the manufacturer’s recommendations. A primer / probe combination specific for the methylated MGMT promoter sequence was used (forward: 5’-GCGTTTCGACGTTCGTAGGT-3’; reverse: 5’-CACTCTTCCGAAAACGAAACG-3’; probe: 5’-6FAM-CGCAAACGATACGCACCGCGA-TMR-3’), with SensiFast Probe Kit (Bioline, Luckenwalde, Germany). Fully methylated CpG Methylase (SssI) treated DNA served as calibrator. The collagenase gene 2A1 (COL2A1), was used as endogenous control (forward: 5’-TCTAACAATTATAAACTCCAACCACCAA-3’; reverse: 5’-GGGAAGATGGGATAGAAGGGAATAT-3’; probe: 5’-6FAM-CCTTCATTCTAACCCAATACCTATCCCACCTCTAAA-TMR-3’). The percentage of methylated reference (PMR) value was calculated by dividing the MGMT / COL2A1 ratio of the sample by the MGMT / COL2A1 ratio of the SssI-treated DNA, and multiplying by 100. Samples with a PMR value > 4 were considered as methylated. All reactions were performed in triplicates.

### Immunohistochemistry analysis of formalin fixed paraffin embedded GBM tissue samples

Immunohistochemistry (IHC) was performed using antibodies specific for nestin (Merck Millipore) and CD15 (immunotools). Slides were processed on an automatic IHC system, AutostainerLink48 (Dako, Hamburg, Germany), following routine protocols.

### Statistics

Statistical analysis was performed using SigmaPlot 10.0 (Systat Software, Inc., Erkrath, Germany). A Mann-Whitney Rank Sum Test was performed to determine significant differences between treatment groups. P values of p<0.05 were considered as significant, p<0.001 was considered as highly significant.

## Results

### Clinically relevant doses of temozolomide increase tumorigenicity of GBM cells *in vitro*

**Fig 1:**
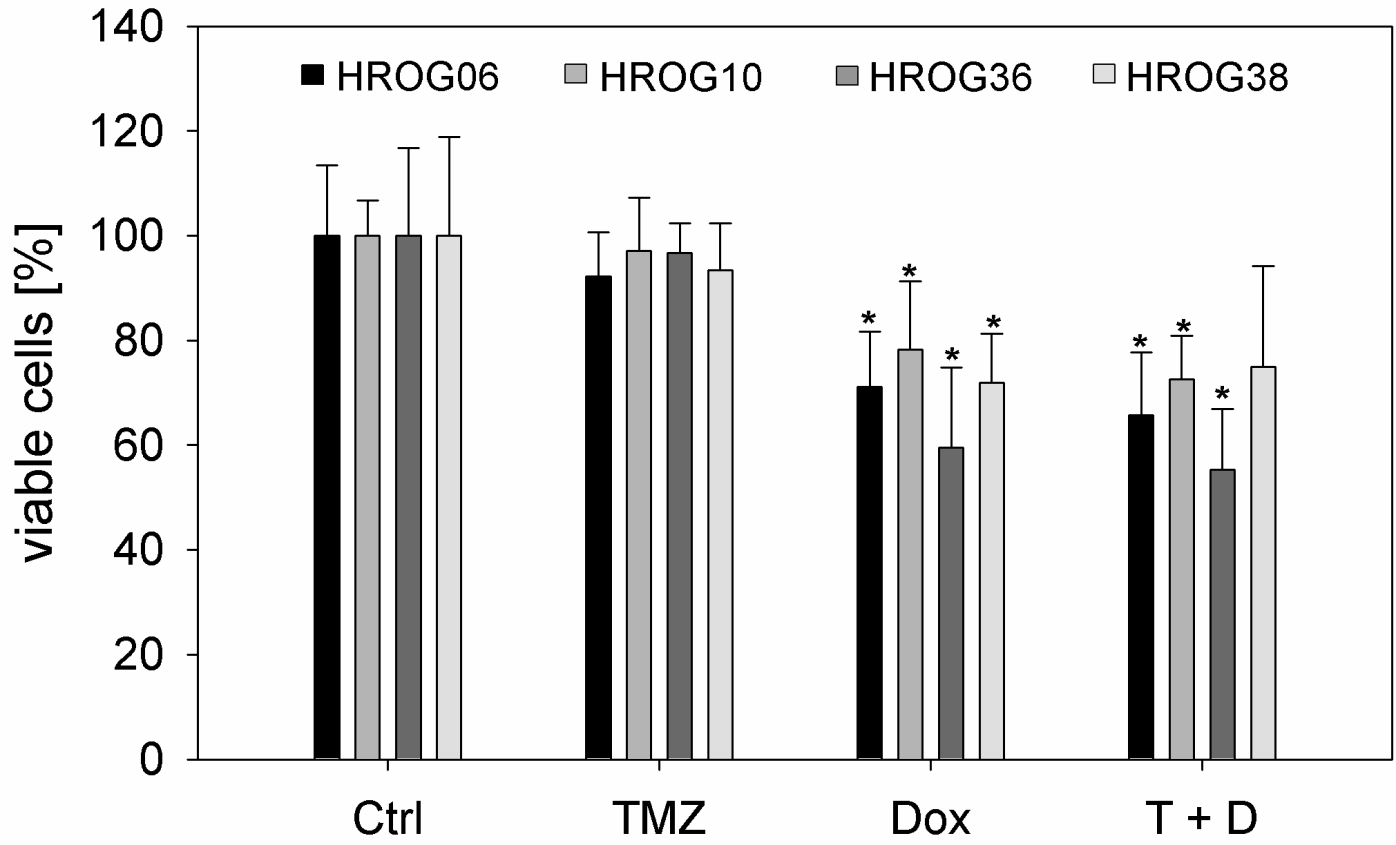
Sensitivity of patient individual GBM cell lines towards TMZ and Dox. Results are given as median values of 4 independent experiments in triplicates. Error bars represent the standard deviation, Ctrl: untreated cells, TMZ: 50μM temozolomide, Dox: 50μM doxycycline, T+D: combination treatment with temozolomide and doxycycline (50μM each), *p<0.05, Mann Whitney Rank sum test.

Four previously in our laboratory established glioblastoma cell lines [16]–HROG06, HROG10, HROG36 and HROG38 –were treated with 50μM TMZ, 50μM Dox or a combination of both substances for 2 cycles of 72h to determine their sensitivity towards this treatment (Fig 1). All cell lines were derived from treatment naïve GBMs and showed an unmethylated MGMT-promoter (S1 Table). TMZ treatment had no effect on the viability of all cell lines at the applied dosis. Dox treatment was able to significantly reduce the amount of viable cells in all cases in comparison to untreated controls. Simultaneous treatment with both substances did not show additional effects compared to Dox monotherapy, indicating that the reduction of viable cells is solely attributable to Dox (Fig 1). The methylation status of the MGMT-promoter remained stable under all treatment conditions (S1 Table).

**Fig 2:**
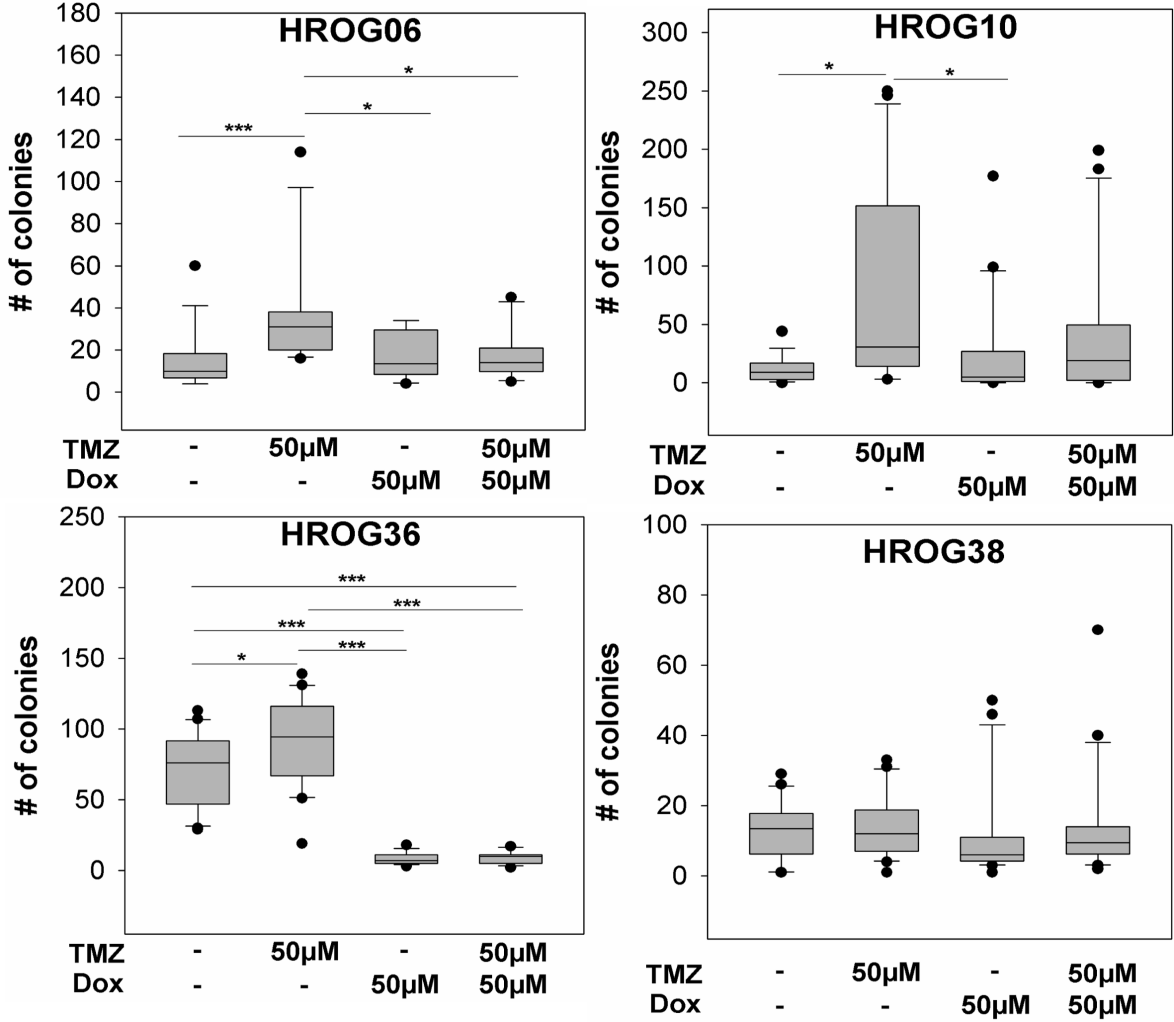
TMZ treatment of non-CSCs leads to increased tumorigenicity *in vitro* which can be diminished by combination treatment with Dox. Tumorigenicity of GBM cell lines after treatment with TMZ, Dox or a combination of both drugs in vitro, * p<0.05, *** p<0.001, Mann Whitney rank sum test.

Surviving cells from each treatment condition and cell line were analyzed further regarding their tumorigenicity *in vitro* by colony formation assays in soft agar. TMZ treated cell lines HROG06, HROG10 and HROG36 showed significantly increased tumorigenicity *in vitro* compared to untreated cells. No difference was observed in case of HROG38 (Fig 2).

### Targeting mitochondria with doxycycline counteracts TMZ induced *in vitro* tumorigenicity

A previous study demonstrated that non-CSCs can (re-)gain CSC properties after TMZ treatment [12]. The increased *in vitro* tumorigenicity after treatment with TMZ might be an indicator for a conversion of GBM cells into a CSC like cell type. Since it has been reported previously that CSCs show an increased dependence on mitochondrial biogenesis, they may be an attractive therapeutic target [14]. In order to determine if simultaneous treatment with Dox can prevent the TMZ induced increase of tumorigenicity *in vitro*, the four GBM cell lines were treated with 50μM Dox and 50μM TMZ alone and in combination. Three cell lines treated with a monotherapy of 50μM Dox showed *in vitro* tumorigenicity levels similar to the untreated controls (HROG06, HROG10 and HROG38), indicating that Dox itself does not influence *in vitro* tumorigenicity in those cell lines (Fig 2). However, in case of the HROG36 cell line, treatment with 50μM Dox alone lead to significantly decreased *in vitro* tumorigenicity compared to untreated controls (p<0.001; Fig 2). Upon combination treatment with TMZ and Dox, the *in vitro* tumorigenicity decreased significantly compared to TMZ treatment in HROG06 and HROG36 (p = 0.004 and p<0.001, respectively; Fig 2). In case of HROG10, we observed a trend towards a decreased *in vitro* tumorigenicity upon combination treatment with TMZ and Dox which did not reach significance (p = 0.066). No treatment effects were observed in case of HROG38 (p = 0.386; Fig 2).

### Expression of GBM-CSC markers nestin and CD15

**Fig 3:**
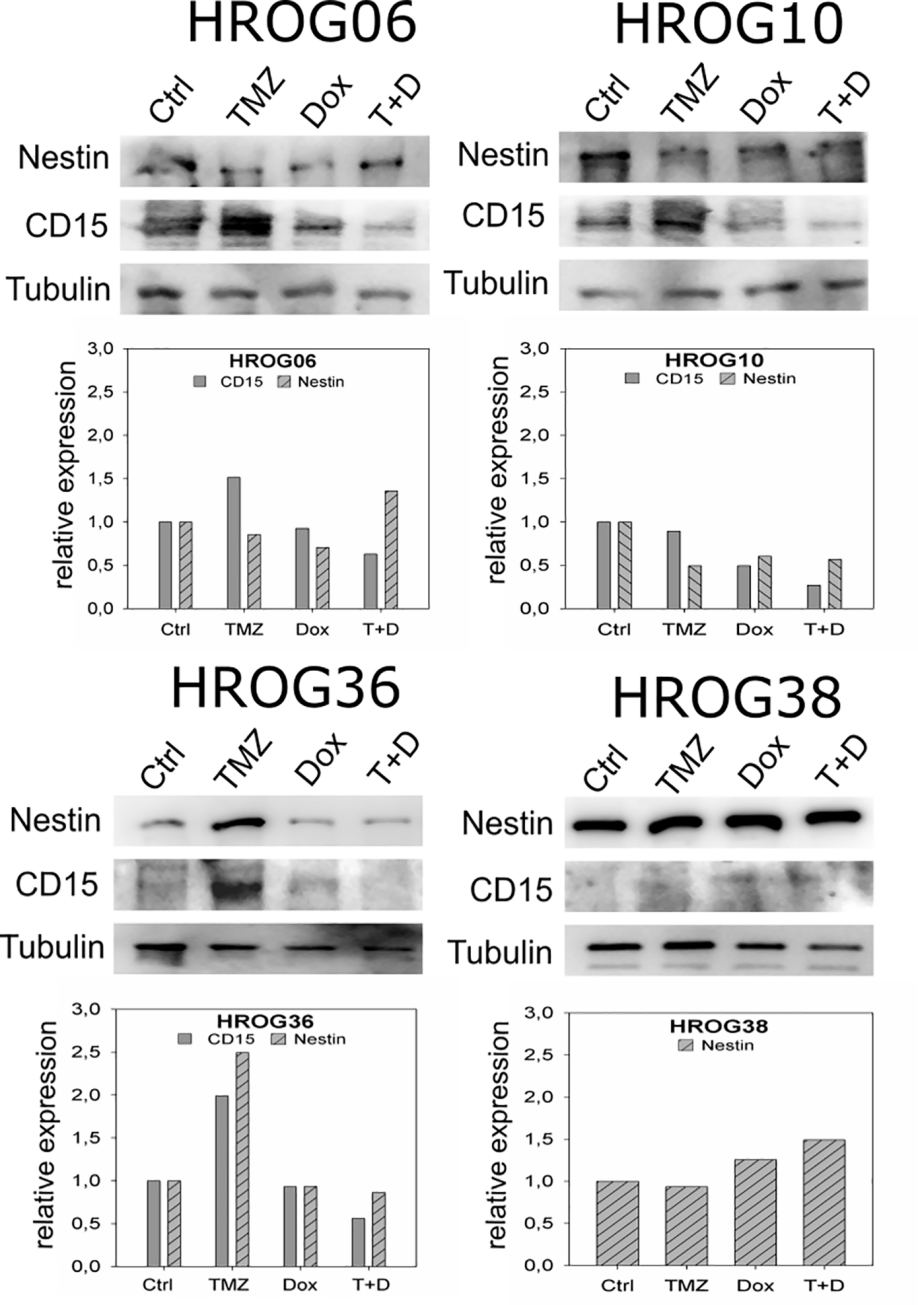
Nestin and CD15 expression after *in vitro* treatment with TMZ, Dox and a combination of both drugs. Upper panels show western blot analysis of GBM non-CSCs under different treatment conditions (50μM TMZ, 50μM Dox or 50μM each), Tubulin represents the loading control. Lower panels are results from densitometric scanning analyses of the western blot signals. Results are given as relative expression to untreated control cells.

GBM tumor cells show increased tumorigenicity *in vitro* after treatment with clinically relevant doses of TMZ. This could possibly be attributable to a conversion of non-CSCs into CSC like cells. Hence, expression of two GBM-CSC markers–CD15 and nestin [9]—was analyzed. CD15 was expressed at low levels (HROG06, HROG10, HROG36) or undetectable (HROG38) in untreated non-CSCs in all four analyzed cell lines. However, increased CD15 expression was observed after treatment with 50μM TMZ compared to untreated cells in HROG06, HROG10 and HROG36 cell lines (Fig 3). All cell lines treated with a combination of 50μM TMZ and 50μM Dox showed expression levels of CD15 comparable to untreated non-CSCs (Fig 3). Expression of the intracellular marker nestin was not affected by TMZ treatment in HROG06, HROG10 and HROG38. In case of HROG36, increased nestin expression was observed after TMZ treatment, but not after Dox or combination treatment (Fig 3).

### Analysis of mitochondria content in GBM cell lines

**Fig 4:**
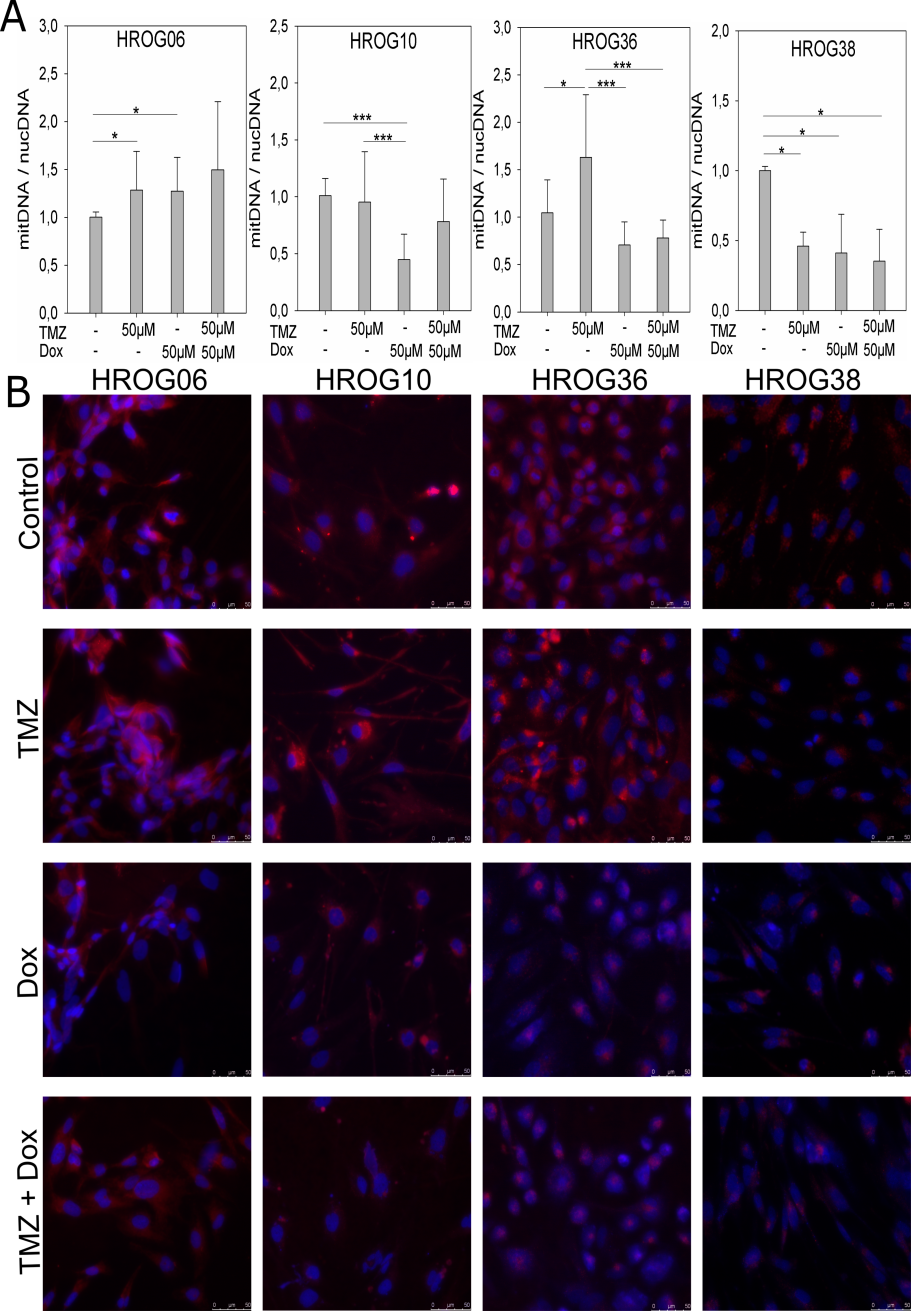
Mitochondria content of GBM non-CSCs can be affected by temozolomide and doxycycline treatment. A) Quantification of mitochondria amount in GBM cell lines via qPCR analysis of mitochondrial DNA in relation to nuclear DNA under different treatment conditions, results are depicted as mean values of 3 independent experiments in triplicates, error bars indicate the standard deviation; * p<0.05, *** p<0.001; B) Fluorescence staining of mitochondria in GBM cell lines under different treatment conditions.

In order to determine the effect of the different treatments on the amount of mitochondria in the GBM cell lines, we quantified the content of mitochondrial DNA in relation to nuclear DNA by qPCR using primer sets specific for mitochondrial DNA or nuclear DNA (Fig 4A). Additionally, mitochondria of all four cell lines treated with 50μM TMZ, 50μM Dox and a combination of both drugs were stained, using the MITO-ID Red Detection Kit (Enzo), and compared to untreated control cells (Fig 4B). In case of HROG06, a significant increase of the amount of mitochondria was observed upon treatment with TMZ and Dox as single agents, but not in combination, in comparison to untreated control cells (TMZ: p = 0.009, Dox: p = 0.017, combination: p = 0.31; Fig 4A). However, microscopic analysis of fluorescence stained mitochondria revealed no marked difference in mitochondria content in HROG06 cells under all treatment regimens and untreated control cells (Fig 4B). In case of HROG10 we observed a significant decrease of mitochondrial DNA by qPCR after Dox treatment compared to untreated control cells (p<0.001) and cells treated with 50μM TMZ (p<0.001; Fig 4A). Direct staining of mitochondria showed a lower staining intensity after treatment with 50μM Dox alone or in combination with 50μM TMZ compared to untreated and TMZ treated cells (Fig 4B). In the HROG36 cell line the amount of mitochondria increased significantly upon treatment with TMZ compared to untreated controls (p = 0.016) in qPCR analysis, which was also observed by fluorescence microscopy of stained mitochondria (Fig 4A and 4B). Additionally we observed a significant decrease of mitochondrial DNA after treatment with Dox (p<0.001) or a combination of TMZ and Dox compared to TMZ treated cells in qPCR analysis (p<0.001; Fig 4A). Again, this effect was clearly visible by fluorescence staining of mitochondria of HROG36 cells (Fig 4B). In case of HROG38 decreased amounts of mitochondrial DNA were evident in all treatment conditions in comparison to untreated controls (p = 0.002), which was confirmed by microscopic analysis (Fig 4B).

### CD15 expression is increased in clinical samples of relapsed GBM

**Fig 5:**
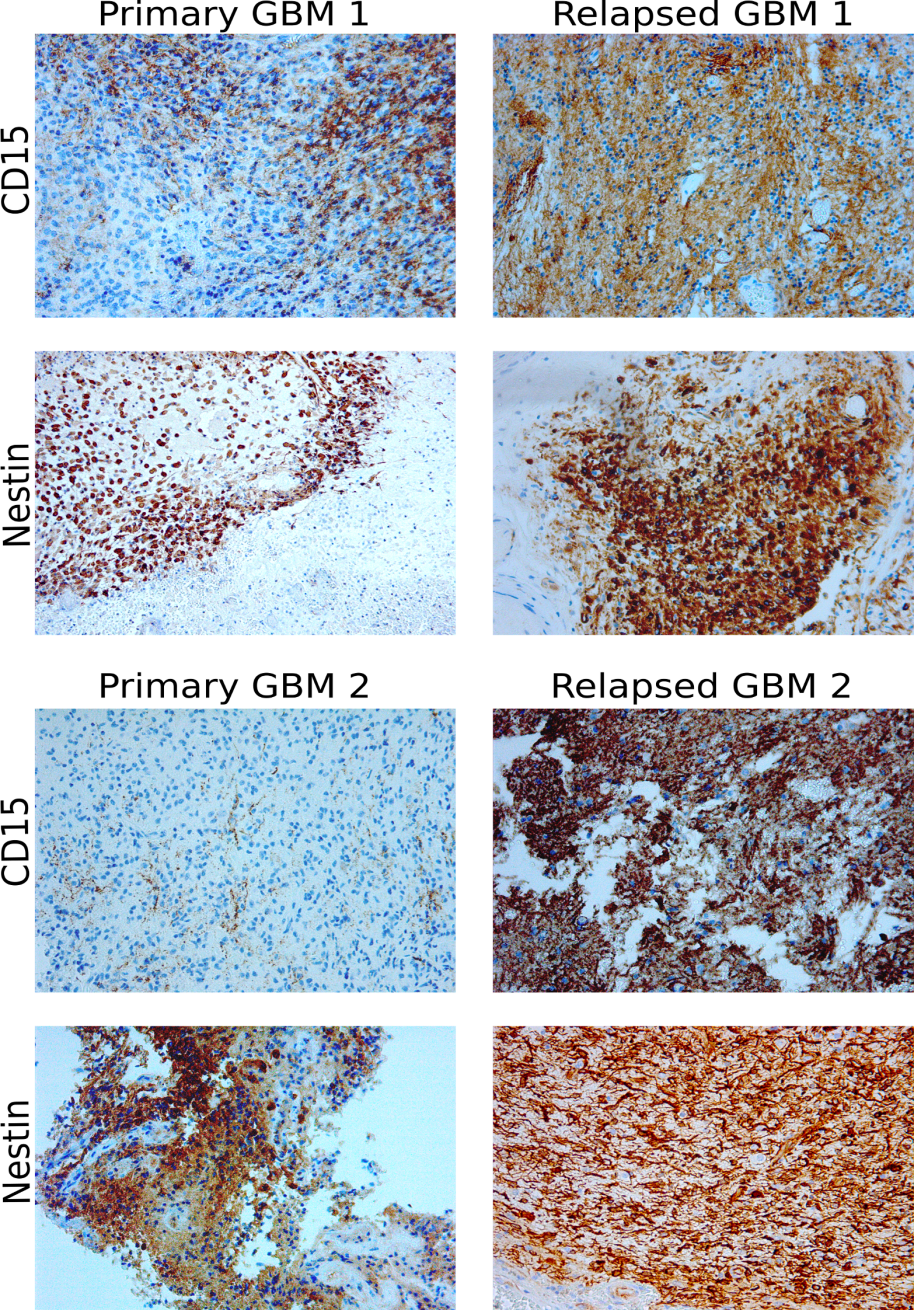
CD15 expression is increased in clinical samples of two relapsed GBM. IHC staining of nestin and CD15 in clinical samples of two cases pre and post chemotherapy with TMZ, 200x magnification.

We could confirm that treatment with 50μM TMZ enhances tumorigenicity *in vitro* and influences the expression of the two GBM CSC markers nestin and CD15. Within our cohort of GBM patients that were operated on at the department of neurosurgery in Rostock, we identified two cases that underwent surgery for both the primary GBM and the relapsed tumor. More importantly, both patients received a TMZ therapy prior to the excision of the relapsed GBM. In the first matched pair (“Primary GBM1” and “Relapsed GBM 1”) TMZ was applied until three days before excision of the relapsed tumor, in case of the second pair (“Primary GBM 2” and “Relapsed GBM2”) the patient received TMZ until 3 months prior to surgery. Formalin fixed paraffin embedded tissue samples of those tumors were analyzed by immunohistochemistry staining of CD15 and nestin (Fig 5, S1 Fig). There were no marked differences in nestin expression in both matched cases. However, increased expression of CD15 was evident in both relapsed GBM samples in comparison to the matched primary GBMs (Fig 5, S1 Fig).

## Discussion

Despite multimodal treatment, GBM remains a lethal tumor with only few long-time survivors [1,2]. Drug resistance is a major challenge of GBM treatment. Several mechanisms of drug resistance, for example increased MGMT expression [21] and upregulation of ABC transporters [11] have been identified for GBM. Another proposed drug resistance mechanism is the loss of cellular hierarchy within a tumor [12], which we explored further in this study. The hypothesis of cellular hierarchy proposes the existence of a small subset of undifferentiated CSCs within a GBM which are capable of self-renewal and tumor growth initiation [6–8]. GBM-CSCs can also differentiate into several neural cell lineages like astrocytes, oligodendrocytes or glia cells, enhancing the tumor mass [22]. However, there is evidence that differentiated tumor cells can undergo dedifferentiation processes to (re-)gain a CSC phenotype and evade chemotherapy [12]. We could detect significantly increased *in vitro* tumorigenicity in 3 out of 4 patient derived GBM cell lines after treatment with clinically relevant TMZ doses (Fig 2), suggesting that TMZ possibly induced a conversion of non-CSCs to a CSC phenotype in those cell lines [12]. This is well in line with the results of the study by Auffinger *et al*., demonstrating conversion of GBM non-CSCs into CSCs after TMZ treatment *in vitro* [12]. However, this effect might also be caused by TMZ induced selection of CSCs in our study. To further investigate the possible conversion of non-CSCs into CSCs we analyzed expression of the two GBM CSC markers nestin and CD15 (Fig 3). We also aimed to target the supposed CSCs by treatment with Dox, because it has been reported that CSCs of several tumor entities–including GBM–show increased dependence on mitochondria [13,14]. The results we obtained lead us to conclude that a potential non-CSC conversion after TMZ therapy is not the only mechanism responsible for increased tumorigenicity *in vitro*. Only one cell line–HROG36 –showed results that were relatively consistent with the hypothesis of loss of cell hierarchy and conversion of non-CSCs into CSCs in our study: 1) increased tumorigenicity after treatment with TMZ that was associated with a higher mitochondria content, 2) diminished tumorigenicity and lowered mitochondria content after Dox treatment and 3) higher expression of both analyzed GBM CSC markers nestin and CD15 after TMZ treatment but not after Dox or combination treatment. In case of HROG06, additional treatment with Dox diminished the TMZ induced increased tumorigenicity, but had no effect on mitochondria content. HROG10 also showed increased tumorigenicity *in vitro* after TMZ treatment, which was not significantly reduced by additional Dox treatment (p = 0.066), despite decreased mitochondria content after Dox treatment in this cell line. Although not investigated further, it seems possible that Dox treatment induced proteotoxic stress in mitochondria causing impaired functionality without decreasing the mitochondria amount [23].

We found increased CD15 expression in those three cell lines that also showed increased *in vitro* tumorigenicity after TMZ treatment (Figs 2 and 3). This result prompted us to analyze CD15 expression by immunohistochemistry staining of FFPE material from 2 patients that underwent surgery for the primary GBM as well as the relapsed tumor. Furthermore both patients received chemotherapy with TMZ until 3 days (case #1) or 3 months (case #2) prior to the second surgery. In both cases, CD15 expression was higher in the relapsed GBMs, whereas the difference in nestin expression was less pronounced (Fig 5, S1 Fig). CD15 is a proposed yet controversial CSC marker for GBM [24,25]. Despite the controversial role of CD15 as a GBM CSC marker surprisingly little is known about its exact function in GBM. CD15 is a member of the family of fucosyltransferases (FUT) that are involved in the generation of fucosylated carbohydrate structures [26]. Furthermore, CD15 promotes proliferation and cell cycle progression via crosstalk with PI3K/Akt and MAPK in A431 cells [27]. Members of the FUT family, including CD15, were linked to multidrug resistance in hepatocellular carcinoma cells via PI3K/Akt signaling [28]. The PI3K/Akt pathway is known to be often deregulated in GBM and might as well be influenced by fucosyltransferases to promote drug resistance [29,30]. There is also evidence of increased expression of fucosylated glycans in other cancers, like breast cancer, lung cancer or colon cancer [31–34]. Thus, analysis of fucosylated glycans and expression of fucosyltransferases could provide more insight in the development of drug resistance [28]. Little is known about the role of fucosyltransferases in drug resistance in GBM. Our *in vitro* data show increased expression of CD15 after chemotherapy with TMZ in those cell lines that also showed increased tumorigenicity after TMZ treatment. Furthermore, we observed increased CD15 expression in two clinical cases in the relapsed GBM in comparison to the respective *de novo* GBMs. Although we could only analyze two clinical cases pre and post chemotherapy with TMZ it seems possible that FUTs are involved in drug resistance and should be further investigated.

Taken together, our data support the conclusion that different resistance mechanisms occur in individual GBM cells *in vitro* and possibly complement one another. A conversion of non-CSCs to cells with a CSC like phenotype seems possible, but appears to be not the only explanation for diminished *in vitro* tumorigenicity upon combination therapy with TMZ and Dox. It is also possible that Dox induced proteotoxic stress in mitochondria of non-CSCs or the possible unspecific inhibition of matrix metalloproteinases by Dox treatment is contributing to the decreased tumorigenicity we observed [35]. Another factor appears to be CD15 whose role in drug resistance development in GBM is largely unknown and our data warrant further research into the influence of fucosyltransferases in GBM.

## Supporting information

S1 Table Patient / cell line characteristics and MGMT-promoter methylation status of the cell lines pre and post treatment with 50μM TMZ, 50μM Dox and a combination of both drugs. (PDF)

S1 Fig Immunohistochemistry staining of several different regions of FFPE material of 2 paired primary and relapsed GBM cases in 100x magnification. (TIF)
