# Supplementary material for: Temozolomide-induced increase of tumorigenicity can be diminished by targeting of mitochondria in in vitro models of patient individual glioblastoma
Source: PLoS One. 2018 Jan 19;13(1):e0191511. doi: 10.1371/journal.pone.0191511 (PMC5774812; doi:10.1371/journal.pone.0191511)
Supplement: S1 Table — (PDF) [file pone.0191511.s001.pdf]

| **Cell line** | **Diagnosis** | **Patient age/gender** | **MGMT-Promotor Methylation** | |
|---|---|---|---|---|
| | | | untreated | treated |
| HROG06 | GBM | 53/M | unmethylated | unmethylated |
| HROG10 | GBM | 74/M | unmethylated | unmethylated |
| HROG36 | GBM | 80/F | unmethylated | unmethylated |
| HROG38 | GBM | 49/F | unmethylated | unmethylated |
