# Supplementary figures and images for: Temozolomide-induced increase of tumorigenicity can be diminished by targeting of mitochondria in in vitro models of patient individual glioblastoma
Source: PLoS One. 2018 Jan 19;13(1):e0191511. doi: 10.1371/journal.pone.0191511 (PMC5774812; doi:10.1371/journal.pone.0191511)

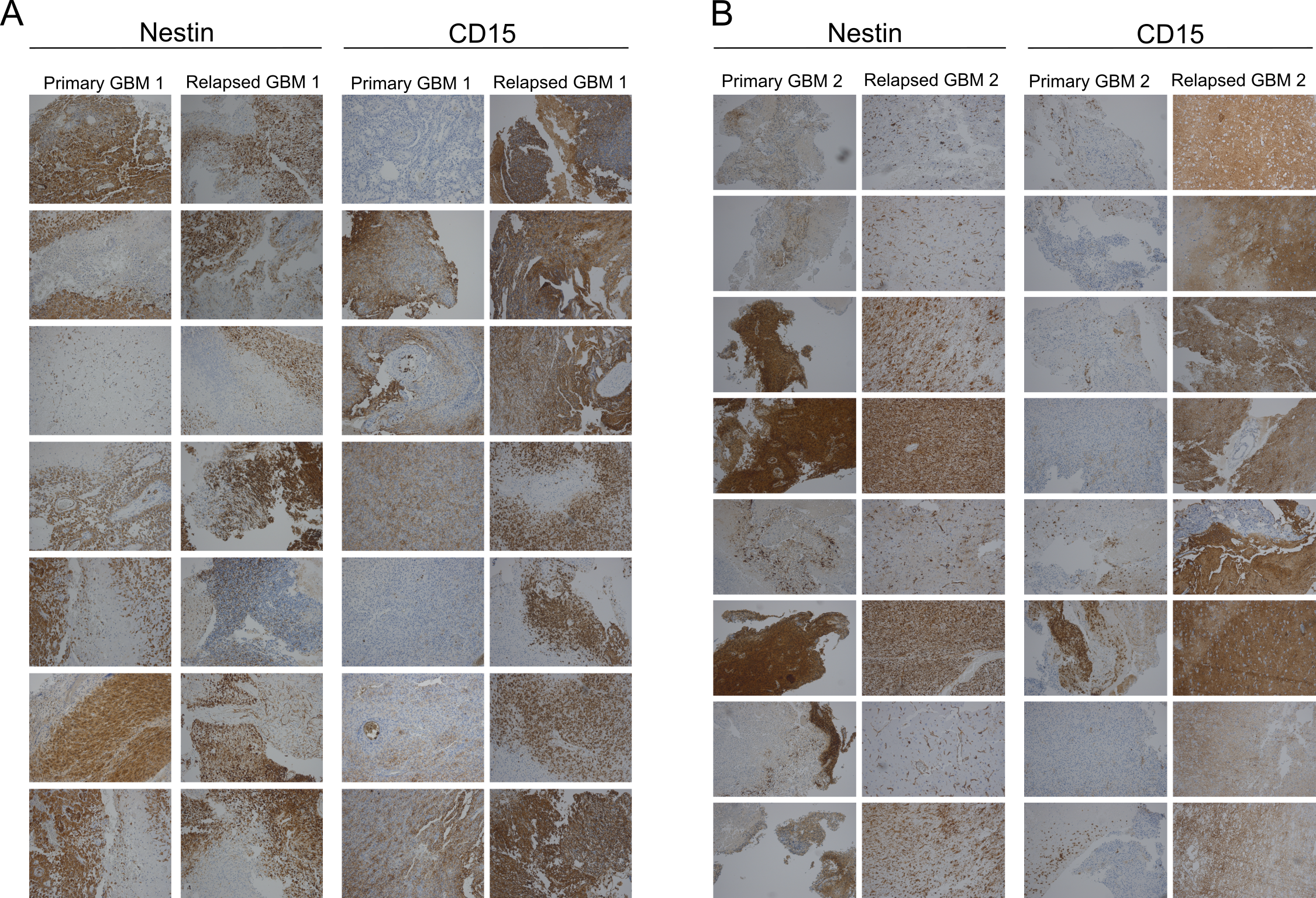

Supplement: S1 Fig — (TIF) [file pone.0191511.s002.tif]
